# Menstrual and reproductive factors associated with risk of breast cancer among Indian women: a cross sectional study from National Family Health Survey, 2019-21

**DOI:** 10.1186/s13690-024-01266-9

**Published:** 2024-04-23

**Authors:** Ujjwal Das, Sabita Soren, Nishamani Kar

**Affiliations:** 1 Dept. of Geography, Rajiv Gandhi University, Itanagar, Arunachal Pradesh India; 2 Dept of Geography, Fakir Mohan University, Balasore, Odisha India; 3 Professor Dept. of Geography, Rajiv Gandhi University, Itanagar, Arunachal Pradesh India

**Keywords:** Breast cancer, Women's menstrual, Reproductive, Odds ratios, Menarche, Breast-feeding

## Abstract

**Background:**

The breast cancer is common cancer in women globally. The risk of breast cancer is strongly associated with women’s menstrual and reproductive factors that have been established in different countries. Therefore present study was aim to explore the association between menstrual and reproductive factors and the risk of breast cancer screening in Indian women.

**Methods:**

The present study data has been used fifth round of the National Family Health Survey (NFHS-V) with 724,115 women in aged 15–49 in 2019-21. The self-reported ever screened of breast cancer for women aged 30–49 was the main outcome variable of the study. Logistic regression models were used to estimate odds ratios and 95% confidence intervals for breast cancer by menstrual and reproductive factors adjusted for potential confounders.

**Results:**

Late menarche (OR = 2.20, 95% CI: 1.48–3.28), irregular menstrual cycle (OR = 1.29, 95% CI: 1.08–3.53)), delay age at first birth (OR = 1.93, 95% CI: 1.11–3.04) and contraceptive pill used (OR = 1.11, 95% CI: 0.74–2.10) were significantly associated to increases the uptake of screening breast cancer. While, a higher number of birth (OR = 0.52, 95% CI: 0.10–1.03), and long duration of breast-feeding practice (OR = 0.75, 95% CI: 0.63–0.91) were reduced to participate for screening breast cancer.

**Conclusion:**

The results of the study confirm the role of menstrual and reproductive factors in breast cancer in Indian women. Therefore, our findings are imperative for developing breast cancer prevention strategies and better preparedness. Creating awareness and providing knowledge on cancer could be key strategies for the reduction of breast cancer in Indian reproductive age group women.

**Supplementary Information:**

The online version contains supplementary material available at 10.1186/s13690-024-01266-9.

**Table:** 

Text box 1 Contributions to the literature
• The late age of menarche (>15 years) was significantly associated with higher screening of breast cancer.
• In India, only one-third of all breast cancer patients present for diagnosis at an early stage.
• The long duration of breastfeeding was significantly reduced the screening of cancer prevalence.
• Women who developed breast screening were more likely to have irregular cycles, had less number of full-term pregnancy and higher body mass index.

## Introduction

Breast cancer is the most common cancer and the leading cause of death for women worldwide [1]. According to the Global Cancer Report, there were 2.2 million new cases of breast cancer worldwide in 2020, accounting for a quarter of cancer cases among women [2]. Epidemiological studies have shown that the global burden of breast cancer is expected to cross almost 2 million by the year 2030 [3]. There was a wide variation of women breast cancer globally. The incidence rate was highest found in northern and western Europe, northern America, Australia, New Zealand, and southern countries of South America, notably Uruguay and Argentina, while in low throughout Asia and Africa [4]. The incidence rate is more than 100 per 100,000 women in several US states, the highest recorded in Montevideo in Uruguay [5]. While, in Asian populations including Hong Kong, Singapore, and the Philippines rates are intermediate such as 30–50 per 100,000 populations [4]. While in country India has one of the highest rates of the most aggressive subtype of BC referred to as Triple Negative Breast Cancer (TNBC) [6]. In India, the incidence rate has significantly increased, almost by 50%, between 1965 and 1985 [7]. The estimated number of incident cases in India in 2016 was 118,000 [8] and will rise up to 200,000 per year by 2030 [9]. Hence, in India, on an average, for every two women newly diagnosed with breast cancer, one is dying from this disease. In 2020; new cases were diagnosed 1, 78,361, and among of them 90,408 death [8]. The screening coverage in the country is lower than the other countries [10]. The existing study mentioned that less than 10% of India women which ranges 25.3 to 48.4% ever undergo breast examination or participate in screening activities [11]. While, other Asian countries like china screening coverage was 57.6–82.3%, Thailand 55.8–63.6% and the Philippines 34.7–51.9% [12]. The lack of awareness, social stigma, familial negligence, out of pocket expenditure, and lack of essential health infrastructure in regional centre are main contributing factors to low coverage of screening rate, late detection and high mortality due to cancer [13]. The previous study mentioned that breast cancer screening was minimal in Andhra Pradesh, where the incidence of breast cancer reported was 48 per 100,000 in the Hyderabad. Similarly, the East Khasi Hills district in Meghalaya had the highest incidence rate [1].The increasing trend was also observed in Bhopal, Chennai and Delhi registries. The study from some developing world shows that socio-economic factors such as age, education, and marital status are important determinants with the likelihood of receiving breast cancer screening [14–16]. A study from Trivandrum, in Kerala found that “Muslim women, unmarried women and those with professional occupation were less likely to undergo clinical breast examination as compared to Hindu women, married women and homemakers” [17]. The record from the National Family Health Survey − 4 in the year 2015-16 that only one in ten women aged between 15 and 49 years had undergone screening for breast cancer [18]. Women are vulnerable section of the population, being disadvantaged both economically and socially, and bear a higher burden of disease [15]. In addition low coverage of screening, increasing trends of breast cancer were observed throughout India, there remains substantial geographic variation in BC incidence across India from a high of 240.8 DALY rate in Kerala to a low of 58.4 DALY rate in Sikkim [19]. And people from rural areas cannot access those facilities and are possibly living with undiagnosed cancer cases, besides, there are large regional variations. The survival rate of breast cancer is poor in India as compared to Western countries due to earlier age at onset, late stage of disease at presentation, delayed initiation of definitive management and inadequate/fragmented treatment [9].

Numerous literatures suggested that many reproductive factors responsible for screening of breast cancer which includes age at menarche, length of menstruation cycle, age at first pregnancy, parity, breast feeding, body mass index, abortion, and smoking status [20–22]. The previous study shows that a high number of menstruation cycle before the full-time pregnancy and high life time menstruation activity increased the risk of breast cancer [23]. The study has also suggested both young age at menarche and late menopause might have exaggerated phase of irregular menstruation cycle and increase breast cancer risk because of the increased number of ovulatory cycles and thus exposure to high estrogen levels for a longer period [22, 24]. The prior study consistently shown that parity and breast feeding were associated with a reduced risk of developing luminal subtype, while spontaneous or induced abortion was related to an increased risk of developing luminal subtype [25]. Apart from that early oral contraceptive use, tobacco use, alcohol use, and family history have been found to increase the risk of breast cancers among the reproductive women in an earlier study [26]. The high body mass index has significantly increased the risk of cancer screening in later age groups [27].

Most of the studies cover socio-economic and intersectional regional variations of women’s breast cancer in the country [12, 28–29]. But empirical evidence of menstruation status and reproductive factors on women’s breast cancer is rare in the country. Of universal concern was that the majority of screening cases in India are late stage which results in poorer breast cancer prognoses [28]. Therefore to fill the gap, this paper examines the association of menstrual and reproductive factors with screening women’s breast cancer in India. This study is important as it maps the target areas and vulnerable groups to improve prognoses, women need to be observed at earlier stages.

## Materials and methods

### Data

The present data have been used in a fifth round of National Family Health (NFHS-5) which was conducted in 2019-21 at the International Institute for Population Sciences, Mumbai under the guidance of the Ministry of Health and Family Welfare (MoHFW), Gov. of India. The NFHS-5 provides reliable information on maternal and child health indicators, nutrition health service utilization, contraceptive use, and disease screening along with socioeconomic and demographic characteristics of households across the country both urban and rural areas. NFHS-5 coverage multistage sampling design like urban areas collected information by census enumeration blocks (CEBs) and villages in rural areas were primary sampling unit (PSUs). Probability Proportional to Size (PPS) sampling was drawn to select the PSUs. In this survey, the questions on screening for and diagnosis of cancer were asked to women aged 15–49 years. This survey comprised a nationally representative sample of 636,699 household, and 724,115 women who were interviewed using a multistage sampling design. The sampling designs of the survey are publically available in the report [30]. The information regarding ever had undergone screening for breast cancer is recommended for the women aged 30 years and above and study have used a sample of 364,556 women age 30–49 years in the analysis.

### Outcome variables

The self-reported breast cancer screening was the main outcome variable of the study. The outcome variable was recorded in the binary formats as ‘yes’ and ‘no’. If the woman ever screened for breast cancer then it’s coded 1 as yes otherwise it’s 0 as no.

#### Independent variables

Menstrual and reproductive factors and covariates were categorized as follows in the study age at menarche (early age such as < 13, 13–15, and delayed age as > 15 year), menopausal status (yes and no), menstrual regularity (regular such as women have periods of 28 days and irregular as fewer than 21 days and more than 35 days apart), number of pregnancies (0, 1, 2, 3, ≥ 4), number of live birth (1, 2, ≥ 3), age at first live birth (< 25, 25–29 and > 29 years), BMI (< 18.5 kg/m2, 18.5 to 24.5 kg/m2 and, > 24.5 kg/m2), terminated pregnancy (Abortion, miscarriage, and stillbirth), oral contraceptive used (yes and no), and smoking status (yes and no).

### Statistical analysis

Descriptive statistics were used in the present study. Unconditional logistic regression models were used to estimate odds ratios (ORs) and their respective 95% confidence intervals (CIs) for associations of breast cancer screening with menstrual and reproductive factors. The multivariable logistic regression model was adjusted for potential confounders based on the literature. The incidence rates of cancer in India were very low and hence, screening proportions were estimated per 100, 000 women. All the statistical analysis was done using the statistical software STATA version 17.0.

## Results

Table 1 shows that the study conducted by NFHS 2019-21 has taken women from the reproductive age group that is between 30 to 49 years of age. The study reveals that most of the women are of moderate BMI and experience a regular menstrual cycle. The highest percentage of women have started first period in age between 13 to 15 years (75.5%). About 81% of women have given their first birth before 25 years of age. With respect to no of live birth, the total 5% of women have no child and 37% of women have two children, only 20% of women have more than three children in the study. Around 72% of women have more than three times of pregnancies. The highest number of women have regular menstruation cycle (98%) and only 2% of women reported from their irregular menstruation cycle. Instead of medical advancement there is a despair picture of contraceptive pills used by women. Only 6% of women are used any type of contraceptive pills in the study. About 61% of women terminated their pregnancy by miscarriages and 31% by abortion respectively. Table A1 of additional file shows the full sample of 15 to 49 years of women.

**Table 1:** Socio-economic and bio-demographic characteristics of study population

Background characteristics	N	%	Background characteristics	N	%
**Age of the respondents**			**Age at 1st birth**		
30–34	1,01,049	27.72	< 25	2,81,006	81.27
35–39	98,068	26.9	25–29	50,918	15.0
40–44	81,380	22.32	> 29	13,825	4.0
45–49	84,059	23.06	**Duration of breast-feeding (Months)**		
**Place of residents**			< 12	15,311	29.75
Urban	95,547	26.21	12–23	15,282	29.69
Rural	2,69,009	73.79	> 24	20,876	40.56
**Marital status**			**Body Mass Index (BMI)**		
Never married	7,663	2.1	< 18.5	36,871	10.43
Married	3,30,345	90.62	18.5–24.9	2,04,373	57.8
Widowed	20,785	5.7	> 25	1,12,357	31.78
Divorced	1,933	0.53	**Menstruation status**		
Separated	3,830	1.05	Irregular	5,042	1.93
**Age of menarche**			Regular	2,56,216	98.07
< 13	46,294	20.16	**Contraceptive pill used**		
13–15	1,73,422	75.52	No	3,44,056	94.0
> 15	9,922	4.32	Yes	20,495	6.0
**No of. Live birth**			**Terminated pregnancy**		
0	19,114	5.24	Abortion	3,287	31.15
1	42,295	11.6	Miscarriage	6,459	61.22
2	1,35,630	37.2	Still birth	805	7.63
3	91,964	25.23	**Smoking status**		
> 3	75,553	20.72	Yes	53,772	14.75
**No. of Pregnancy**			No	3,10,784	85.25
1	185	3.66			
2	529	10.47			
3	681	13.48			
> 3	3,657	72.39			

Table 2 shows the menstrual and reproductive factors variation in the proportion of breast cancer screening per 100, 000 women aged 30–49 years. The proportion of cancer screening was higher among the women whose started their first menstruation cycle at delayed age > 15 years. The proportion of cancer screening were significantly higher among women with delayed age at first birth (*P* < 0.001), terminated the pregnancy by miscarriage and abortion (*P* < 0.001), high body mass index (*P* < 0.001), and use more contraceptive pills (*P* < 0.001) respectively. The proportion of breast cancer screening increased with women age, being 530 for aged 30–34 and 600 for 35–39 years. Women who are used smoked had a higher proportion of screening breast cancer at without significant level (*P* = 0.151).

**Table 2:** Menstrual and reproductive factors associated with screening of breast cancer among women age 30–49 years (Per 100,000 women) in India

Background characteristics	Screening Breast cancer	Without Screening Breast cancer	λ^2^ (P)	Background characteristics	Screening Breast cancer	Without Screening Breast cancer	λ^2^ (P)
**Age of menarche**				**Maternal age**			
< 13	180	99,820	10.35	30–34	530	99,470	
13–15	190	99,810	*P* = *0.006*	35–39	600	99,400	*34.69*
> 15	330	99,670		40–44	740	99,260	*P* < 0.001
**Menstruation status**				45–49	690	99,310	
Irregular	710	99,290	2.07	**Age at 1st birth**			
Regular	600	99,400	*P* = 0.047	< 25	600	99,400	
**Menopause started**				25–29	740	99,260	36.74
No	640	99,360	7.26	> 29	950	99,050	*P* < 0.001
Yes	510	99,490	*P* = 0.007	**Contraceptive pill used**			
**No of. Live birth**				No	370	99,630	48.58
0	650	99,350		Yes	570	99,430	*P* < 0.001
1	780	99,220	192.55	**Duration of breast-feeding (Months)**			
2	820	99,180	*P* < 0.001	< 12	570	99,430	
3	520	99,480		12–24	570	99,430	8.78
> 3	360	99,640		> 24	380	99,620	*P* = 0.012
**No. of Pregnancy**				**Body Mass Index (BMI)**			
1	1640	98,360		< 18.5	440	99,560	
2	950	99,050	2.96	18.5–24.9	490	99,510	259.76
3	450	99,550	*P* = 0.397	> 25	950	99,050	*P* < 0.001
> 3	750	99,250		**Smoking status**			
**Terminated pregnancy**				No	340	99,660	3.78
Abortion	500	99,500		Yes	640	99,370	*P* = 0.151
Miscarriage	900	99,100	7.24				
Still birth	250	99,750	*P* < 0.001				

Table 3 shows crude and adjusted ORs and 95% CIs for breast cancer by women menstrual and reproductive factors. After adjustment for potential confounders, women who reached early and late age at menarche was significantly associated with higher screening of breast cancer risk (OR = 1.15, 95% CI: 1.05–2.38 and OR = 2.20, 95% CI: 1.48–3.28) compared to women menarche age 13–15 years. Women menopause was associated non-significantly elevated screening of breast cancer compared to those women not reached menopause. The women irregular menstrual cycle had 1.29 time (95% CI: 1.08–3.53) increases the screening of breast cancer. Compared to women with lower child, women with three or more child had low screening breast cancer (OR = 0.52, 95% CI: 0.10–1.03). Women who had given first birth at the age of above 29 years had significantly 1.93 times (95% CI: 1.11–3.04) higher chances of screening breast cancer as compared to those women given birth at below 25 years of age. The duration of breast feeding more than 24 months had reduced 25% chances of screening breast cancer (OR = 0.75, 95% CI: 0.63–0.91). The screening of breast cancer were significantly higher among those women had used the contraceptive pill (OR = 1.11, 95% CI: 0.70–2.10) and terminated their pregnancy by the abortion (OR = 1.63, 95% CI: 0.99–2.69).

**Table 3:** Screening of breast cancer (Odd Ratios) among women with their menstrual and reproductive factors

	Crude OR (95% CI)		Adjusted OR^a^ (95% CI)	
Background characteristics	OR	95% CI	OR	95% CI
**Age of menarche**				
13–15®				
< 13	1.10**	1.02–2.39	1.15	1.05–2.38
> 15	1.85***	1.25–2.85	2.20***	1.48–3.28
**Menstruation status**				
Regular®				
Irregular	1.25**	1.00–2.56	1.29***	1.08–3.53
**Menopause started**				
No®				
Yes	1.55	1.08–2.36	1.69*	1.15–3.38
**No. of. Live birth**				
0®				
1	2.88***	2.54–3.27	2.75	2.41–3.69
2	3.66***	3.27–4.09	3.30*	2.69–4.52
3	2.60***	2.29–2.96	2.45**	2.12–3.20
> 3	1.88***	1.62–2.19	1.12***	1.05–2.36
**No. of Pregnancy**				
1®				
2	0.77	0.32–1.88	0.47	0.04–5.82
3	0.67	0.28–1.63	0.36	0.03–5.02
> 3	0.73	0.34–1.57	0.30	0.02–3.69
**Age at 1st birth**				
< 25®				
25–29	1.24***	1.1–1.49	1.25	1.01–2.55
> 29	1.92***	1.52–2.17	1.93**	1.11–3.04
**Duration of breast-feeding**				
< 12®				
12–23 months	1.01	0.45–2.28	1.06	0.67–2.16
> 24 months	0.75***	0.63–0.91	0.95***	0.42–2.10
**Contraceptive pill used**				
No®				
Yes	1.65***	0.58–2.74	1.11**	0.74–2.10
**Terminated pregnancy**				
Miscarriage®				
Abortion	1.78***	1.25–2.55	1.63***	0.99–2.69
Still birth	0.53	0.21–1.33	0.70	0.25–1.95

OR: odds ratio; CI: confidence interval

^a^Adjusted for age, place of residence, Wealth status, BMI and smoking status

***p < 0.01 **p < 0.05 *p < 0.1; ® Reference category

## Discussion

The purpose of the study was to explore the association between menstrual and reproductive factors with the screening of breast cancer among Indian women aged 30–49 years. The results of the study showed that early age at menarche (< 13 years), and late age of menarche (> 15 years) were significantly associated with higher screening of breast cancer. This finding consisting’s of the previous studies that showed young age at menarche is associated with an increased screening of breast cancer because of the luminal A subtype [31]. “Early-onset of menarche was related to early and greater cumulative exposure to estrogen, in which the presence of progesterone can increase the risk of breast cancer, particularly in the luminal A subtype in which estrogen exposure is most relevant” [32]. Another meta-analysis result showed that every year increased 1.05 times screening of breast cancer due to early age at menarche among young women [33]. The screening of breast cancer are more pronounced in those women who have reached the menopause phase without a significant level. The prior study mentioned that the uptake of screening were significantly higher in women with later age of menopause compared to women with an earlier age at menopause [34]. The possible explanation is that late menopause is associated with longer duration and increased lifetime exposure to estrogen, which may account for the increased screening of breast cancer [34]. The study found that screening uptake was significantly higher among women in their irregular cycles. Although some studies have shown irregular menstrual cycles not elevated women to increasing their breast screening [35]. Because the “irregular menstrual cycle elated to anovulation, leading to reduced exposure to estrogen and progesterone, and thus, lower screening of breast cancer” [36]. While others study have found no association between screenings of cancer with irregular menstruation cycles [37]. There was no evidence in our data that women’s menstrual cycle a proportionately greater in the luteal phase and associated with breast cancer. The existing study mentioned that longer cycles (greater than 28 days) were not associated with reduced screening rate, while shorter cycles (less than 28 days) appeared to reduce the screening of breast cancer, rather than increase [38].

Our findings reveal that those women were lower numbers of pregnancies and live birth were significantly more likely to participate in any level of breast screening process. These findings existing an earlier study, that the number of pregnancy play a potential effect on breast cancer such as reducing the estrogen and progesterone hormones and increasing the sex hormone-binding globulin, which is reduced the risk of breast cancer screening [39]. Another study indicates that in the pre-menopausal period, each full-term pregnancy leads to a 3% reduction in breast cancer and it reaches 12% in post-menopausal women [40]. And those women who had more than three children had a lower rates of breast screening as compared to women with one child. Furthermore, those women who had given birth at exact age 30 years had significantly lower participate for breast screening as compared to those who are given first birth after the age of 35 years. The earlier study shows “increasing age was significantly associated with the uptake of undergoing breast examination” [41]. The longer duration of breastfeeding practices (> 24 months) has a protective effect on breast screening. The previous study mentioned that every 12 months of breastfeeding reduced the 4.3% breast examination [42]. An interesting result was found those women were terminated pregnancies by abortion had a higher tendency of breast screening as compared to those who were stillbirth. The study also found higher contraceptives using women had the highest coverage of breast screening. Cultural and religious beliefs often interweave to form distinctive traditions and rules that affect women’s decision to participate in screening [43]. Age at starting oral contraceptive use might be determinant in increasing breast screening [44]. A higher level of BMI (> 24.5 kg/m^2^), was a positive predictor of participation in screening among the reproductive age group of women. An increased body fat mass has been associated with early puberty and menarche. The previous study has suggested that increasing the BMI level increases the testosterone which is the influence of elevated androgens with polycystic ovary syndrome and those women who are overweight have higher levels of these hormones even in the absence of polycystic ovary syndrome [44].

## Conclusion

In conclusion, the study demonstrated that menstrual and reproductive factors are significantly associated with women breast screening among the reproductive group of women ages 30–49 years. The study explores that changes in patterns of current lifestyle might have resulted in early and late age at menarche, late marriage at first birth, declined pregnancy, and live birth which play an important role in the development of breast screening among Indian women. Therefore, our findings are imperative for developing breast cancer prevention strategies and better preparedness. A robust awareness campaign and effective implementation of a national cancer screening program are the need of the hour. Furthermore, a high-quality national screening program for women’s cancer with high coverage and participation and an effective referral system is very much required to reduce the cancer prevalence of Indian women.

## Limitations and strengths of the study

Our study has some limitations. Firstly the present study was based on self-reported disease information by women which could be recall bias. Secondly, the age group of the study is restricted to 15–49 years with emphasis on 30–49 because the NFHS provides data for this age group only. Thirdly, the data does not provide information on the family history of breast cancer, which may be a significant association of uptake breast screening in the next generation that was proved in earlier studies [45]. Apart from the limitations, the study has a large sample size, and standardized data collection processes are significant strengths of the study. Moreover, this is the first epidemiological study to investigate the relationship between women’s menstrual cycle with reproductive factors and uptake of breast cancer screening in the country.

### Electronic supplementary material

Below is the link to the electronic supplementary material.

Supplementary Material 1

## Data Availability

The data is publicly available from https://dhsprogram.com/data/dataset/India_Standard-DHS_2020.cfm?fag=0.

## References

[1] Ginsburg O, Rositch AF, Conteh L, Mutebi M, Paskett ED, Subramanian S (2018). Breast cancer disparities among women in low-and middle-income countries. Curr Breast Cancer Rep.

[2] Weiss JM, Lacey Jr JV, Shu XO, Ji BT, Hou L, Yang G, Zheng W (2008). Menstrual and reproductive factors in association with lung cancer in female lifetime nonsmokers. Am J Epidemiol.

[3] DeSantis C, Siegel R, Bandi P, Jemal A (2011). Breast cancer statistics, 2011. Cancer J Clin.

[4] Bray F, McCarron P, Parkin DM. (2004). The changing global patterns of female breast cancer incidence and mortality. *Breast cancer research*, *6*(6), 1–11. https://DOI10.1186/bcr932. 10.1186/bcr932 PMC1064079 15535852

[5] Peto R, Boreham J, Clarke M, Davies C, Beral V (2000). UK and USA breast cancer deaths down 25% in year 2000 at ages 20–69 years. Lancet.

[6] Yeole BB, Kurkure AP (2003). An epidemiological assessment of increasing incidence and trends in breast cancer in Mumbai and other sites in India, during the last two decades. Asian Pac J Cancer Prev.

[7] Saxena, S., Szabo, C. I., Chopin, S., Barjhoux, L., Sinilnikova, O., Lenoir, G.,…Bhatanager, D. (2002). BRCA1 and BRCA2 in Indian breast cancer patients. *Human mutation*, *20*(6), 473–474. https://DOI:10.1002/humu.9082. 10.1002/humu.9082 12442273

[8] Mehrotra R, Yadav K (2022). Breast cancer in India: Present scenario and the challenges ahead. World J Clin Oncol.

[9] International Agency for Research on Cancer. India Source: Globocan 2020. [cited 11 June 2021] Available from: https://gco.iarc.fr/today/data/factsheets/populations/356-india-fact-sheets.pdf.

[10] Leong SP, Shen ZZ, Liu TJ, Agarwal G, Tajima T, Paik NS, Foulkes WD (2010). Is breast cancer the same disease in Asian and western countries?. World J Surg.

[11] Patil P, Sarang B, Bhandarkar P, Ghoshal R, Roy N, Gadgil A (2023). Does women’s empowerment and their socioeconomic condition affect the uptake of breast cancer screening? Findings from NFHS-5, India. BMC Womens Health.

[12] Negi J, Nambiar D (2021). Intersectional social-economic inequalities in breast cancer screening in India: analysis of the National Family Health Survey. BMC Womens Health.

[13] Pati S, Hussain MA, Chauhan AS, Mallick D, Nayak S (2013). Patient navigation pathway and barriers to treatment seeking in cancer in India: a qualitative inquiry. Cancer Epidemiol.

[14] Gopika MG, Prabhu PR, Thulaseedharan JV (2022). Status of cancer screening in India: an alarm signal from the National Family Health Survey (NFHS-5). J Family Med Prim Care.

[15] Islam RM, Billah B, Hossain MN, Oldroyd J (2017). Barriers to cervical cancer and breast cancer screening uptake in low-income and middle-income countries: a systematic review. Asian Pac J cancer Prevention: APJCP.

[16] Kulkarni SV, Mishra GA, Dusane RR. Determinants of compliance to breast cancer screening and referral in low socio-economic regions of urban India. Int J Prev Med. 2019;10. 10.4103/ijpvm.IJPVM_335_17. 10.4103/ijpvm.IJPVM_335_17 PMC6547800 31198519

[17] Frie, K. G., Ramadas, K., Anju, G., Mathew, B. S., Muwonge, R., Sauvaget, C.,… Sankaranarayanan,R. (2013). Determinants of participation in a breast cancer screening trial in Trivandrum district, India. *Asian Pacific Journal of Cancer Prevention*, *14*(12), 7301–7307. 10.7314/apjcp.2013.14.12.7301 24460292

[18] Ministry of Health and Family, Welfare G. National Family Health Survey (NFHS-4). In. Mumbai: IIPS; 2015–6.

[19] Dhillon PK (2018). The burden of cancers and their variations across the States of India: the global burden of disease study 1990–2016. Lancet Oncol.

[20] Sreedevi A, Quereshi MA, Kurian B, Kamalamma L (2014). Screening for breast cancer in a low middle income country: predictors in a rural area of Kerala, India. Asian Pac J Cancer Prev.

[21] Bodapati SL, Babu GR (2013). Oncologist perspectives on breast cancer screening in India-results from a qualitative study in Andhra Pradesh. Asian Pac J Cancer Prev.

[22] Ghose A, Basak S, Agarwal T (2022). Self-breast examination for breast cancer screening: the Indian story. Indian J Cancer.

[23] Olsson HL, Olsson ML (2020). The menstrual cycle and risk of breast cancer: a review. Front Oncol.

[24] Wallace RB, Sherman BM, Bean JA, Leeper JP, Treloar AE (1978). Menstrual cycle patterns and breast cancer risk factors. Cancer Res.

[25] Lambertini, M., Santoro, L., Del Mastro, L., Nguyen, B., Livraghi, L., Ugolini, D.,… Azim Jr, H. A. (2016). Reproductive behaviors and risk of developing breast cancer according to tumor subtype: A systematic review and meta-analysis of epidemiological studies. *Cancer treatment reviews*, *49*, 65–76. https://doi:10.1016/j.ctrv.2016.07.006. 10.1016/j.ctrv.2016.07.006 27529149

[26] Harris HR, Titus LJ, Cramer DW, Terry KL (2017). Long and irregular menstrual cycles, polycystic ovary syndrome, and ovarian cancer risk in a population-based case‐control study. Int J Cancer.

[27] Ganesan DK, Krishnan GK, Chitharaj RR, Boopathirajan R. (2019). A cross-sectional study on relationship between body mass index and menstrual irregularity among rural women in Tamil Nadu.

[28] Changkun Z, Bishwajit G, Ji L, Tang S (2022). Sociodemographic correlates of cervix, breast and oral cancer screening among Indian women. PLoS ONE.

[29] Sen S, Khan PK, Wadasadawala T, Mohanty SK (2022). Socio-economic and regional variation in breast and cervical cancer screening among Indian women of reproductive age: a study from National Family Health Survey, 2019-21. BMC Cancer.

[30] IIPS ICF. National Family Health Survey-5 (2019-21), India Report. 2022. Available from: https://dhsprogram.com/pubs/pdf/FR375/FR375.pdf.

[31] Bui, O. T., Tran, H. T., Nguyen, S. M., Dao, T. V., Bui, Q. V., Pham, A. T.,… Shu,X. O. (2022). Menstrual and reproductive factors in association with breast cancer risk in Vietnamese women: a case-control study. *Cancer Control*, *29*, https://DOI:10.1177/10732748221140206. 10.1177/10732748221140206 PMC9663618 36373740

[32] Beral V, Bull D, Doll R, Peto R, Reeves G, van den Brandt PA, Goldbohm RA (2004). Collaborative group on hormonal factors in breast cancer: breast cancer and abortion: collaborative reanalysis of data from 53 epidemiological studies, including 83000 women with breast cancer from 16 countries. Lancet.

[33] Li H, Sun X, Miller E, Wang Q, Tao P, Liu L, Li J (2017). BMI, reproductive factors, and breast cancer molecular subtypes: a case-control study and meta-analysis. J Epidemiol.

[34] Cirillo PM, Wang ET, Cedars MI, Chen LM, Cohn BA (2016). Irregular menses predicts ovarian cancer: prospective evidence from the Child Health and Development studies. Int J Cancer.

[35] Brinton LA, Moghissi KS, Westhoff CL, Lamb EJ, Scoccia B (2010). Cancer risk among infertile women with androgen excess or menstrual disorders (including polycystic ovary syndrome). Fertil Steril.

[36] Vandeloo MJ, Bruckers LM, Janssens JP. (2007). Effects of lifestyle on the onset of puberty as determinant for breast cancer. Eur J Cancer Prev, 17–25. 10.1097/01.cej.0000220635.38847.6e 17220700

[37] Lee E, Ma H, McKean-Cowdin R, Van Den Berg D, Bernstein L, Henderson BE, Ursin G (2008). Effect of reproductive factors and oral contraceptives on breast cancer risk in BRCA1/2 mutation carriers and noncarriers: results from a population-based study. Cancer Epidemiol Biomarkers Prev.

[38] Clavel-Chapelon F, Gerber M (2002). Reproductive factors and breast cancer risk. Do they differ according to age at diagnosis?. Breast Cancer Res Treat.

[39] Russo J, Moral R, Balogh GA, Mailo D, Russo IH (2005). The protective role of pregnancy in breast cancer. Breast Cancer Res.

[40] Collaborative Group on Hormonal Factors in Breast Cancer. Breast cancer and breastfeeding: collaborative reanalysis of individual data from 47 epidemiological studies in 30 countries, including 50 302 women with breast cancer and 96 973 women without the disease. Lancet. 2002;360(9328):187–95. 10.1016/S0140-6736(02)09454-0. 10.1016/S0140-6736(02)09454-0 12133652

[41] Guo J, Huang Y, Yang L, Xie Z, Song S, Yin J, Qin W (2015). Association between abortion and breast cancer: an updated systematic review and meta-analysis based on prospective studies. Cancer Causes Control.

[42] Tam CS, de Zegher F, Garnett SP, Baur LA, Cowell CT (2006). Opposing influences of prenatal and postnatal growth on the timing of menarche. J Clin Endocrinol Metabolism.

[43] Vahabi M, Lofters A, Kim E, Wong JPH, Ellison L, Graves E, Glazier RH (2017). Breast cancer screening utilization among women from muslim majority countries in Ontario, Canada. Prev Med.

[44] Nagata C, Wada K, Nakamura K, Hayashi M, Takeda N, Yasuda K (2011). Associations of body size and reproductive factors with circulating levels of sex hormones and prolactin in premenopausal Japanese women. Cancer Causes Control.

[45] Shiyanbola OO, Arao RF, Miglioretti DL, Sprague BL, Hampton JM, Stout NK, Trentham-Dietz A (2017). Emerging trends in family history of breast cancer and associated risk. Cancer Epidemiol Biomarkers Prev.

