# Supplementary material for: Menstrual and reproductive factors associated with risk of breast cancer among Indian women: a cross sectional study from National Family Health Survey, 2019-21
Source: Arch Public Health. 2024 Apr 23;82:55. doi: 10.1186/s13690-024-01266-9 (PMC11041000; doi:10.1186/s13690-024-01266-9)
Supplement: Supplementary file 1 — Supplementary Material 1 [file 13690_2024_1266_MOESM1_ESM.docx]

**Table: A1** Sample characteristics of the study women aged 15-49 years, India, 2019-21.

| **Background characteristics** | **N** | **%** | **Background characteristics** | **N** | **%** |
| --- | --- | --- | --- | --- | --- |
| **Age of the respondents** | | | **No. of Pregnancy** | |  |
| 15-19 | 122480 | 16.91 | 1 | 1109 | 3.91 |
| 20-24 | 118700 | 16.39 | 2 | 3267 | 11.51 |
| 25-29 | 118379 | 16.35 | 3 | 3997 | 14.09 |
| 30-34 | 101049 | 13.95 | >3 | 20001 | 70.49 |
| 35-39 | 98068 | 13.54 | **Age at 1^st^ birth** | |  |
| 40-44 | 81380 | 11.24 | <25 | 418710 | 84.76 |
| 45-49 | 84059 | 11.61 | 25-29 | 61484 | 12.45 |
| **Place of residents** | |  | >29 | 13825 | 2.8 |
| Urban | 95,547 | 26.21 | **Duration of breast-feeding (Months)** | | |
| Rural | 269,009 | 73.79 | <12 | 61218 | 36.86 |
| **Marital status** | |  | 12-24 | 52249 | 31.46 |
| Never married | 7,663 | 2.10 | >24 | 52618 | 31.68 |
| Married | 330,345 | 90.62 | **Body Mass Index (BMI)** | | |
| Widowed | 20,785 | 5.70 | <18.5 | 124989 | 17.87 |
| Divorced | 1933 | 0.53 | 18.5-24.9 | 420415 | 60.11 |
| Separated | 3830 | 1.05 | >25 | 153958 | 22.01 |
| **Age of menarche** | |  | **Menstruation status** | |  |
| <13 | 46294 | 19.32 | Irregular | 16807 | 3.05 |
| 13-15 | 183422 | 76.54 | Regular | 533549 | 96.95 |
| >15 | 9922 | 4.14 | **Contraceptive pill used** | | |
| **No of. Live birth** | |  | No | 469832 | 85.38 |
| 0 | 220613 | 30.47 | Yes | 80441 | 14.62 |
| 1 | 108511 | 14.99 | **Terminated pregnancy** | | |
| 2 | 199254 | 27.52 | Abortion | 7696 | 25.91 |
| 3 | 113560 | 15.68 | Miscarriage | 19548 | 65.81 |
| >3 | 82177 | 11.35 | Still birth | 2458 | 8.28 |
|  |  |  | **Smoking status** | |  |
|  |  |  | Yes | 184794 | 25.52 |
|  |  |  | No | 539141 | 74.48 |
